# Supplementary material for: Movement of accessible plasma membrane cholesterol by the GRAMD1 lipid transfer protein complex
Source: eLife. 2019 Nov 14;8:e51401. doi: 10.7554/eLife.51401 (PMC6905856; doi:10.7554/eLife.51401)
Supplement: Supplementary file 1. [file elife-51401-supp1.docx]

| **Key Resources Table** | | | | |
| --- | --- | --- | --- | --- |
| **Reagent type (species) or resource** | **Designation** | **Source or reference** | **Identifiers** | **Additional information** |
| strain, strain background *(E. coli)* | BL21(DE3) Rosetta T1R | Sigma-Aldrich/Merck | B2935 | Chemically competent |
| cell line (*Cercopithecus aethiops*) | COS-7 | Other | YS72 | Gift from Min Wu |
| cell line (*Homo sapiens*) | HeLa M | Other | YS45 | Gift from Pietro De Camilli |
| cell line (*Homo sapiens*) | GRAMD1-TKO #15 | This paper | TNC32 | GRAMD1a/ GRAMD1b/ GRAMD1c Triple Knock Out cell line (See Materials and Methods) |
| antibody | Anti-GRAMD1a  (Rabbit polyclonal) | BETHYL | RRID:AB_2621409 | WB (1:1000) |
| antibody | Anti-GRAMD1B  (Rabbit polyclonal) | Proteintech | 24905-1-AP | WB (1:1000) |
| antibody | Anti-c-myc  (Mouse monoclonal) | Abcam | RRID:AB_1947601 | WB (1:1000) |
| antibody | Anti-CD44  (Mouse monoclonal) | CST | RRID:AB_10547133 | WB (1:1000) |
| antibody | Anti-EEA1  (Rabbit polyclonal) | Thermo | RRID:AB_2096819 | WB (1:1000) |
| antibody | Anti-VAPA  (Rabbit polyclonal) | Sigma | RRID:AB_1080549 | WB (1:1000) |
| antibody | Anti-VAPB  (Rabbit polyclonal) | Sigma | RRID:AB_1858717 | WB (1:1000) |
| antibody | Anti-Actin  (Mouse monoclonal) | EMD Millipore | RRID:AB_2223041 | WB (1:1000) |
| antibody | Anti-SREBP-2  (Mouse monoclonal) | Santa Cruz Biotechnology | RRID:AB_2194250 | WB (1:500) |
| antibody | GFP-Trap | Chromotek | RRID:AB_2631357 |  |
| antibody | Myc-Trap | Chromotek | RRID:AB_2631369 |  |
| antibody | Goat Anti-Rabbit IgG (H+L)-HRP Conjugate | Bio Rad | RRID:AB_11125142 | WB (1:5000) |
| antibody | Goat Anti-Mouse IgG (H+L)-HRP Conjugate | Bio Rad | RRID:AB_11125547 | WB (1:5000) |
| recombinant DNA reagent | EGFP-linker-luminal He (plasmid) | This paper | BE2 | See Materials and Methods |
| recombinant DNA reagent | EGFP-linker-luminal He with 5E (plasmid) | This paper | BE3 | See Materials and Methods |
| recombinant DNA reagent | pNIC28-Bsa4 GRAMD1a StART L366-S537 (plasmid) | This paper | BE4 | See Materials and Methods |
| recombinant DNA reagent | pNIC28-Bsa4 GRAMD1a StART L366-S537 5P (plasmid) | This paper | BE12 | See Materials and Methods |
| recombinant DNA reagent | pNIC28-Bsa4 GRAMD1b StART Q375-E545 (plasmid) | This paper | BE5 | See Materials and Methods |
| recombinant DNA reagent | pNIC28-Bsa4 GRAMD1b StART Q375-E545 5P (plasmid) | This paper | BE9 | See Materials and Methods |
| recombinant DNA reagent | pNIC28-Bsa4 GRAMD1b StART Q375-E545 T469D  (plasmid) | This paper | BE10 | See Materials and Methods |
| recombinant DNA reagent | pNIC28-Bsa4 GRAMD1c StART L325-I500 (plasmid) | This paper | BE18 | See Materials and Methods |
| recombinant DNA reagent | pNIC28-Bsa4 GRAMD1a GRAM M81-E220 (plasmid) | This paper | BE38 | See Materials and Methods |
| recombinant DNA reagent | pNIC28-Bsa4 GRAMD1b GRAM G70-D231(plasmid) | This paper | BE37 | See Materials and Methods |
| recombinant DNA reagent | EGFP-D4 -CLOPF-ec01 (plasmid) | This paper | BE64 | See Materials and Methods |
| recombinant DNA reagent | pNIC28-Bsa4 EGFP-D4H (plasmid) | This paper | BE66 | See Materials and Methods |
| recombinant DNA reagent | EGFP-GRAMD1a (plasmid) | This paper | LK20 | See Materials and Methods |
| recombinant DNA reagent | EGFP-GRAMD1a GRAM (81 - 220) (EGFP-GRAM_1a_) (plasmid) | This paper | D1 | See Materials and Methods |
| recombinant DNA reagent | EGFP-GRAMD1b (plasmid) | This paper | LK21 | See Materials and Methods |
| recombinant DNA reagent | EGFP-GRAMD1b GRAM (92 - 207) (EGFP-GRAM_1b_) (plasmid) | This paper | D2 | See Materials and Methods |
| recombinant DNA reagent | EGFP-GRAMD1b (ΔGRAM) (plasmid) | This paper | LK71 | See Materials and Methods |
| recombinant DNA reagent | EGFP-GRAMD1b (4E) (plasmid) | This paper | TN17 | See Materials and Methods |
| recombinant DNA reagent | EGFP-GRAMD1b (5E) (plasmid) | This paper | TN22 | See Materials and Methods |
| recombinant DNA reagent | EGFP-GRAMD1b (ΔHelix)  (plasmid) | This paper | TN18 | See Materials and Methods |
| recombinant DNA reagent | EGFP-GRAMD1b (TM swap) (plasmid) | This paper | TN92 | See Materials and Methods |
| recombinant DNA reagent | EGFP-GRAMD1c  (plasmid) | This paper | LK22 | See Materials and Methods |
| recombinant DNA reagent | EGFP-GRAMD1c GRAM (65 -186) (EGFP-GRAM_1c_)  (plasmid) | This paper | D4 | See Materials and Methods |
| recombinant DNA reagent | EGFP-GRAMD3  (plasmid) | This paper | DD6 | See Materials and Methods |
| recombinant DNA reagent | mCherry-GRAMD3  (plasmid) | This paper | DD5 | See Materials and Methods |
| recombinant DNA reagent | Myc-GRAMD1a  (plasmid) | This paper | LK69 | See Materials and Methods |
| recombinant DNA reagent | Myc-GRAMD1b  (plasmid) | This paper | LK28 | See Materials and Methods |
| recombinant DNA reagent | Myc-GRAMD3 (plasmid) | This paper | DD7 | See Materials and Methods |
| recombinant DNA reagent | PX459-GRAMD1A_V2_Front (plasmid) | This paper | LK43 | See Materials and Methods |
| recombinant DNA reagent | PX459-GRAMD1B_Back (plasmid) | This paper | LK45 | See Materials and Methods |
| recombinant DNA reagent | PX459-GRAMD1c_sgRNA_#1 (plasmid) | This paper | TN10 | See Materials and Methods |
| recombinant DNA reagent | PX459-GRAMD1c_sgRNA_#2 (plasmid) | This paper | TN11 | See Materials and Methods |
| recombinant DNA reagent | mRuby-GRAMD1a  (plasmid) | This paper | TN28 | See Materials and Methods |
| recombinant DNA reagent | mRuby-GRAMD1b  (plasmid) | This paper | LK26 | See Materials and Methods |
| recombinant DNA reagent | mRuby-GRAMD1b (5P)  (plasmid) | This paper | TN44 | See Materials and Methods |
| recombinant DNA reagent | mRuby-GRAMD1b (T469D)  (plasmid) | This paper | TN40 | See Materials and Methods |
| recombinant DNA reagent | mRuby-GRAMD1b (Y430A, V445A) (plasmid) | This paper | TN42 | See Materials and Methods |
| recombinant DNA reagent | mRuby-GRAMD1b (TM swap) (plasmid) | This paper | TN93 | See Materials and Methods |
| recombinant DNA reagent | mRuby-GRAMD1c  (plasmid) | This paper | TN29 | See Materials and Methods |
| recombinant DNA reagent | mRuby-OSBP  (plasmid) | This paper | D42 | See Materials and Methods |
| recombinant DNA reagent | mRuby-ORP4  (plasmid) | This paper | D35 | See Materials and Methods |
| recombinant DNA reagent | mRuby-ORP9  (plasmid) | This paper | D39 | See Materials and Methods |
| recombinant DNA reagent | mCherry-STARD4 (plasmid) | This paper | TN135 | See Materials and Methods |
| recombinant DNA reagent | PM-FRB-mCherry (plasmid) | This paper | TN133 | See Materials and Methods |
| recombinant DNA reagent | mCherry-pMagFas2(x3)-GRAMD1b(164-738) (plasmid) | This paper | TN27 | See Materials and Methods |
| recombinant DNA reagent | miRFP-pMagFas2(x3)-GRAMD1b(164-738) (plasmid) | This paper | TN56 | See Materials and Methods |
| recombinant DNA reagent | miRFP-FKBP-GRAMD1b (WT) (plasmid) | This paper | TN60 | See Materials and Methods |
| recombinant DNA reagent | miRFP-FKBP-GRAMD1b (5P) (plasmid) | This paper | TN67 | See Materials and Methods |
| recombinant DNA reagent | miRFP-FKBP-GRAMD1b (T469D) (plasmid) | This paper | TN65 | See Materials and Methods |
| recombinant DNA reagent | pMyc-C1  (plasmid) | Other | YS281 | Gift from Pietro De Camilli  PMID: 23791178 |
| recombinant DNA reagent | iRFP-PH^PLCδ^  (plasmid) | Other | YS174 | Gift from Pietro De Camilli  PMID: 22847441 |
| recombinant DNA reagent | mCherry-pMagFast2(3x)-MTMR1  (plasmid) | Other | YS349 | Gift from Pietro De Camilli  PMID: 29463750 |
| recombinant DNA reagent | RFP-Sec61β  (plasmid) | Other | YS134 | Gift from Pietro De Camilli  PMID: 18442980 |
| recombinant DNA reagent | mCherry-LactC2  (plasmid) | Other | YS182 | Gift from Pietro De Camilli |
| recombinant DNA reagent | mRuby-C1  (plasmid) | Addgene | #54552 |  |
| recombinant DNA reagent | PM-FRB-CFP  (plasmid) | Addgene | #67517 |  |
| recombinant DNA reagent | pSpCas9(BB)-2A-Puro (PX459) V2.0  (plasmid) | Addgene | #62988 |  |
| recombinant DNA reagent | mCherry-FKBP-MTM1  (plasmid) | Addgene | #51614 |  |
| chemical compound, drug | (DOPC) 1,2-dioleoyl-sn-glycero-3-phosphocholine | Avanti | 850375 |  |
| chemical compound, drug | (Dansyl-PE) 1,2-dioleoyl-sn-glycero-3-phosphoethanolamine-N-(5-dimethylamino-1-naphthalenesulfonyl) | Avanti | 810330 |  |
| chemical compound, drug | (DHE) ergosta-5,7,9(11),22-tetraen-3ß-ol | Avanti | 810253 |  |
| chemical compound, drug | 16:0-18:1 PE (POPE) 1-palmitoyl-2-oleoyl-sn-glycero-3-phosphoethanolamine | Avanti | 850757C |  |
| chemical compound, drug | 18:1 PA 1,2-dioleoyl-sn-glycero-3-phosphate (sodium salt) | Avanti | 840875C |  |
| chemical compound, drug | 18:1 PS (DOPS) 1,2-dioleoyl-sn-glycero-3-phospho-L-serine (sodium salt) | Avanti | 840035C |  |
| chemical compound, drug | 18:1 SM (d18:1/18:1(9Z)) N-oleoyl-D-erythrosphingosylphosphorylcholine | Avanti | 860587C |  |
| chemical compound, drug | PI(4)P diC16 Phosphatidylinositol 4-phosphate diC16 | Echelon | P-4016 |  |
| chemical compound, drug | Brain PI(4,5)P2 | Avanti | 840046X |  |
| chemical compound, drug | 4ME 16:0 PC 1,2-diphytanoyl-sn-glycero-3-phosphocholine | Avanti | 850356C |  |
| chemical compound, drug | 16:0-18:1 PC (POPC) 1-palmitoyl-2-oleoyl-glycero-3-phosphocholine | Avanti | 850457C |  |
| chemical compound, drug | Cholesterol Sigma Grade | Sigma-Aldrich/Merck | C8667-5G |  |
| chemical compound, drug | Cholesterol / MCD | This paper | Brown et al, Mol. Cell 2002 |  |
| chemical compound, drug | Complete, EDTA-free Protease inhibitors | Sigma-Aldrich/Merck | 11873580001 |  |
| chemical compound, drug | Co-TALON® Metal Affinity Resin | Takara | 635503 |  |
| chemical compound, drug | Gel filtration calibration kit LMW | GE healthcare | 28-4038-41 |  |
| chemical compound, drug | Benzonase® Nuclease | Sigma-Aldrich/Merck / Santa Cruz Biotechonology | E1014-25KU / sc-202391 |  |
| chemical compound, drug | Deoxyribonuclease I crude lypholized | Sigma-Aldrich/Merck | DN25-100MG |  |
| chemical compound, drug | Lysozyme from hen egg white | Sigma-Aldrich/Merck | 62970-1G-F |  |
| chemical compound, drug | IPTG | Thermo scientific | R0393 |  |
| chemical compound, drug | HisPur Ni-NTA Resin | Thermo scientific | 88222 |  |
| chemical compound, drug | Glycerol Ultrapure, 99% Nuclease Free, MB Grade | Affymetrix/ USB Products | US16374-1L |  |
| chemical compound, drug | DMEM(4.5g/l G lucose) with L-Gln, without Sodium Pyruvate | Nacalai Tesque, Japan | 08459-35 |  |
| chemical compound, drug | Fetal Bovine Serum. Origin: EU Approved South American | Gibco | 10270-106 |  |
| chemical compound, drug | Lipofectamine 2000 Reagent | Invitrogen | 11668-019 |  |
| chemical compound, drug | Lipoprotein Deficient Serum from fetal calf (LPDS) | Sigma-Aldrich/Merck | S5394-50ML |  |
| chemical compound, drug | Methyl-B-cyclodextrin, Cell culture tested | Sigma-Aldrich/Merck | C4555-5G |  |
| chemical compound, drug | Mevastatin | Santa Cruz Biotechnology | sc-200853 |  |
| chemical compound, drug | Opti-MEM™ I Reduced Serum Medium | Thermo scientific | 31985070 |  |
| chemical compound, drug | Puromycin | Stemcell | 73342 |  |
| chemical compound, drug | Q5 High-Fidelity DNA Polymerase | NEB | M0491S |  |
| chemical compound, drug | Rapamycin | Sigma-Aldrich/Merck | R0395 |  |
| chemical compound, drug | Sphingomyelinase from Bacillus cereus (SMase) | Sigma-Aldrich/Merck | S9396 |  |
| commercial assay or kit | Colloidal blue staining kit | Thermo scientific | LC6025 |  |
| commercial assay or kit | BCA Protein Assay Kit | Invitrogen | 23225 |  |
| commercial assay or kit | NEBuilder HiFi DNA Assembly Cloning Kit | NEB | E5520S |  |
| commercial assay or kit | Q5 Site-Directed Mutagenesis Kit | NEB | E0552S |  |
| commercial assay or kit | Quickextract™ DNA Extraction Solution 1.0 | Lucigen | LUCG-QE09050 |  |
| commercial assay or kit | ZERO BLUNT FOR SEQ | Invitrogen | 450031 |  |
| software, algorithm | GraphPad Prism | GraphPad Software | RRID:SCR_002798 |  |
| software, algorithm | Fiji | Fiji | RRID:SCR_002285 |  |
