# Supplementary material for: Movement of accessible plasma membrane cholesterol by the GRAMD1 lipid transfer protein complex
Source: eLife. 2019 Nov 14;8:e51401. doi: 10.7554/eLife.51401 (PMC6905856; doi:10.7554/eLife.51401)
Supplement: Supplementary file 2. — A list of sequence-based reagents. DNA sequences for oligos and primers used in this study are described. [file elife-51401-supp2.docx]

| **Reagent type (species) or resource** | **Designation** | **Source or reference** | **Identifiers** | **Additional information** |
| --- | --- | --- | --- | --- |
| sequence-based reagent | EGFP-GRAMD1b 674-718aa  (gBlock) | This paper | BEP-1 | GAGATATACCATGGGGATGGTTAGTAAAGGTGAGGAACTGTTCACAGGGGTGGTCCCCATTCTTGTGGAGTTAGATGGAGATGTGAACGGACATAAATTCAGCGTAAGTGGCGAGGGCGAGGGCGACGCCACCTACGGAAAATTGACCCTTAAATTCATTTGCACTACTGGAAAATTGCCAGTCCCGTGGCCTACATTGGTCACCACCCTGACTTACGGAGTGCAGTGCTTCTCTCGCTACCCTGACCACATGAAACAACACGACTTCTTTAAGTCCGCTATGCCTGAGGGCTACGTGCAGGAACGCACGATTTTTTTCAAAGATGACGGCAATTACAAGACCCGCGCAGAAGTTAAATTTGAGGGCGACACACTGGTAAATCGCATCGAATTAAAGGGAATTGACTTTAAAGAGGACGGAAACATTCTTGGTCATAAACTGGAGTATAATTACAATAGTCACAATGTATATATCATGGCCGACAAACAGAAAAATGGGATTAAGGTTAATTTTAAAATTCGTCATAACATTGAAGACGGCTCTGTGCAATTAGCAGACCATTACCAGCAAAATACACCGATCGGAGACGGCCCTGTTTTACTTCCTGATAACCATTACTTATCCACGCAGAGTGCCCTTAGTAAGGACCCAAACGAGAAACGTGATCATATGGTATTACTGGAGTTCGTCACGGCTGCTGGCATTACGCTTGGTATGGATGAATTATATAAGGGTGGAGGCGGTTCCGGCGGAGGTGGCTCCGGCGGTGGCGGATCCTCTCAGACCGAGTGGGCGCAGTTGTTGGAATCCCAACAAAAGTATCACGATACCGAACTGCAAAAATGGCGTGAAATCATTAAAAGTAGCGTGATGTTGCTGGATCAAATGAAAGACTCCTTGATTAATTTACAGCTCGAGCACCACCA |
| sequence-based reagent | 5'NcoI_EGFP_1b_Helix  (primers) | This paper | BEP-2 | GAGATATACCATGGGGATGGTTAGTAAAGGTGAGGAACTG |
| sequence-based reagent | 3'XhoI_eGFP_1b_Helix  (primers) | This paper | BEP-3 | TGGTGGTGCT  CGAGCTGTAA  ATTAATCAAGG  AGTCTTTC |
| sequence-based reagent | EGFP-GRAMD1b_Helix_5E  (gBlock) | This paper | BEP-4 | GCCACCTACGGAAAATTGACCCTTAAATTCATTTGCACTACTGGAAAATTGCCAGTCCCGTGGCCTACATTGGTCACCACCCTGACTTACGGAGTGCAGTGCTTCTCTCGCTACCCTGACCACATGAAACAACACGACTTCTTTAAGTCCGCTATGCCTGAGGGCTACGTGCAGGAACGCACGATTTTTTTCAAAGATGACGGCAATTACAAGACCCGCGCAGAAGTTAAATTTGAGGGCGACACACTGGTAAATCGCATCGAATTAAAGGGAATTGACTTTAAAGAGGACGGAAACATTCTTGGTCATAAACTGGAGTATAATTACAATAGTCACAATGTATATATCATGGCCGACAAACAGAAAAATGGGATTAAGGTTAATTTTAAAATTCGTCATAACATTGAAGACGGCTCTGTGCAATTAGCAGACCATTACCAGCAAAATACACCGATCGGAGACGGCCCTGTTTTACTTCCTGATAACCATTACTTATCCACGCAGAGTGCCCTTAGTAAGGACCCAAACGAGAAACGTGATCATATGGTATTACTGGAGTTCGTCACGGCTGCTGGCATTACGCTTGGTATGGATGAATTATATAAGGGTGGAGGCGGTTCCGGCGGAGGTGGCTCCGGCGGTGGCGGATCCTCTCAAACAGAATGGGCACAACTTCTGGAGTCGCAGCAAAAGTATCACGATACCGAGGAACAAAAAGAACGTGAAGAGGAAAAGTCCAGTGTGATGTTGGAGGACCAAATGAAAGATTCGTTAATCAATTTACAGCTCGAGCACCACCA |
| sequence-based reagent | GRAMD1a_I429SNPL_F  (primers) | This paper | BEP-13 | accaccaGGCCCCAAGAGCGCCTCC |
| sequence-based reagent | GRAMD1a_I429SNPL_R  (primers) | This paper | BEP-14 | ggtggtggGGGGATGGTGTACGTCAGCAC |
| sequence-based reagent | D4_D434S_F  (primers) | This paper | BEP-104 | AAATTATCAAtcaAAAACAGCTCACTATTCAAC |
| sequence-based reagent | D4_D434S_R  (primers) | This paper | BEP-105 | CCATCCCATGTTTTATGAG |
| sequence-based reagent | 3'_KpnI-stop_GRAMD1b_683aa  (primers) | This paper | TNP-33 | GGGCCCGCGGTACCTCAGTACTTTTGTTGGGACTCTAAGAGC |
| sequence-based reagent | 3’ KpnI D4_Mam_Stop  (primers) | This paper | LK016 | GGGCCCGCGGTACCTTAATTGTAGGTTATGGAACTACCTG |
| sequence-based reagent | 5’ EcoRI D4_Mam  (primers) | This paper | LK015 | CAAGCTTCGAATTCAAAGGGGAAAATCAATTTGGACCATT |
| sequence-based reagent | 3'-BamHI-stop-GRAMD1b-C-AS  (primers) | This paper | TNP-55 | TCTAGATCCGGTGGATCCTCAATGATAGCGATTCCTCTTTTCTTC |
| sequence-based reagent | Genotyping_1B_F2  (primers) | This paper | LK164 | GACTGTGCTCATATTAGCCAGAGACCTAAGTGC |
| sequence-based reagent | Genotyping_1B_R1  (primers) | This paper | LK165 | CAGAGTGGTGGGTGAGCCTCATAAGGCTTCGG |
| sequence-based reagent | GRAMD1a _Stop_R  (primers) | This paper | LK215 | GCCCGCGGTACCTCAGGAAAAGCTGTCATCGGGCCGGGGC |
| sequence-based reagent | GRAMD1a_F  (primers) | This paper | LK214 | CTCAGATCTCGAGAAATGTTCGACACCACACCCCACTCTG |
| sequence-based reagent | GRAMD1a_GRAM Domain_F  (primers) | This paper | DKP-29 | GTCCGGACTCAGATCTCGAGAAATGCTGAGCCCCACTTATAAG |
| sequence-based reagent | GRAMD1a_GRAM Domain_R  (primers) | This paper | DKP-30 | GTGGATCCCGGGCCCGCGGTACCTCACTCTGAGCCGTAGCACTG |
| sequence-based reagent | GRAMD1a_Genotyping_V2V3_F2  (primers) | This paper | TNP-9 | AAAGGACTGGGGTGTGGCCTCCTCATG |
| sequence-based reagent | GRAMD1a_Genotyping_V2V3_R2  (primers) | This paper | TNP-11 | TTGAGGCTCAAACACCCTCTGTAGATG |
| sequence-based reagent | GRAMD1a-sgRNA#1_AS  (primers) | This paper | LK186 | AAACTGCGTCAGCACCTCGGAGTCC |
| sequence-based reagent | GRAMD1a-sgRNA#1_S  (primers) | This paper | LK185 | CACCGGACTCCGAGGTGCTGACGCA |
| sequence-based reagent | GRAMD1b DeltaNterm F  (primers) | This paper | LK035 | CAGATCTCGAGAAACAGCTCGCCTCATTCCCAATGCCATCCAAGTTTGC |
| sequence-based reagent | GRAMD1b_434LTNPL_F  (primers) | This paper | TNP-73 | TCCTCCTGCTCCCAAAACTGCCACT |
| sequence-based reagent | GRAMD1b_434LTNPL_R  (primers) | This paper | TNP-74 | GGAGGAGGGGTGATGGTGTAAAGAATCAC |
| sequence-based reagent | GRAMD1b_F  (primers) | This paper | LK216 | TCAGATCTCGAGAAATGAAAGGATTCAAGCTCTCCTGCAC |
| sequence-based reagent | GRAMD1b_Short GRAM Domain_F  (primers) | This paper | DKP-31 | GGACTCAGATCTCGAGAAAAGCAGAGAAATGAAGACTTCAGAAAGC |
| sequence-based reagent | GRAMD1b_Short GRAM Domain_R  (primers) | This paper | DKP-32 | GGATCCCGGGCCCGCGGTACCTCACTTTTCAAGGAGAGCATTCTGC |
| sequence-based reagent | GRAMD1b_Helix_2_5E_F  (primers) | This paper | TNP-49 | GAAATCCTCAGTGATGCTCGAGATCAACCTTCAGAACGGC |
| sequence-based reagent | GRAMD1b_Helix_2_5E_R  (primers) | This paper | TNP-50 | TCCTCTTCCCTCTCTTTTTGCTCCTCAGTATCGTGGTACTTTTG |
| sequence-based reagent | GRAMD1b_Helix4E_F  (primers) | This paper | TNP-44 | GTCCCAACAAAAGGAACACGATACTGAGCTCCAAAAATG |
| sequence-based reagent | GRAMD1b_Helix4E_R  (primers) | This paper | TNP-45 | TCTTCTTCCTGGGCCTCTTCTGTCTGAGACTGGGG |
| sequence-based reagent | GRAMD1b_Stop_R  (primers) | This paper | LK217 | GGCCCGCGGTACCTCAATGATAGCGATTCCTCTTTTCTT |
| sequence-based reagent | GRAMD1b_T469D_F  (primers) | This paper | TNP-57 | CGAAGTCCTCGACCACGACGTG |
| sequence-based reagent | GRAMD1b_T469D_R  (primers) | This paper | TNP-58 | GCATCTATCACGTAACATTC |
| sequence-based reagent | GRAMD1b_Y430A_V445A_F  (primers) | This paper | TNP-77 | CTGGCTCCCAAAACTGCCACTGCCAGGGAGACACAGACCATG |
| sequence-based reagent | GRAMD1b_Y430A_V445A_R  (primers) | This paper | TNP-78 | AGGGTTGGTAAGGGTGATGGTGGCAAGAATCACTCGGCTCTG |
| sequence-based reagent | GRAM of GRAMD1B Rev  (primers) | This paper | LK223 | CCGCCGTACCTCATTTTTCTTTAGTCATGGAACAGATGTCTTTCAAACGG |
| sequence-based reagent | 5'_GRAMD1b_iEcoRV_Fw_HiFi  (primers) | This paper | TNP-107 | AGCAGCGGCGCTTCTCTGATATC |
| sequence-based reagent | 3'_GRAMD1b_621_Rv_HiFi  (primers) | This paper | TNP-108 | GGACACGCTCTGCAGGTGGAAAC |
| sequence-based reagent | 5'_GRAMD1b-Sec61b_TM_Fw_HiFi  (primers) | This paper | TNP-109 | TCCACCTGCAGAGCGTGTCCAAAGTTGGCCCTGTTCCAGTATTG |
| sequence-based reagent | 3'_pEGFP_Sec61b_TM_Rv_HiFI  (primers) | This paper | TNP-110 | GGTGGATCCCGGGCCCGCGGTACCCTACGAACGAGTGTACTTGC |
| sequence-based reagent | GRAMD1b-sgRNA#1_AS  (primers) | This paper | LK154 | AAACCACGGGTGAGCGTGTAGCGAC |
| sequence-based reagent | GRAMD1b-sgRNA#1_S  (primers) | This paper | LK153 | CACCGTCGCTACACGCTCACCCGTG |
| sequence-based reagent | GRAMD1c_F  (primers) | This paper | LK218 | CTCAGATCTCGAGAAATGGAGGGCGCTCCGACTGTCCGTC |
| sequence-based reagent | GRAMD1c_GRAM Domain_F  (primers) | This paper | DKP-35 | CAAGTCCGGACTCAGATCTCGAGAAAAAGACAGGAATGAGGAATAC |
| sequence-based reagent | GRAMD1c_GRAM Domain_R  (primers) | This paper | DKP-36 | GATCCCGGGCCCGCGGTACCCTATTCCTGTCTAGTCAGGCTC |
| sequence-based reagent | GRAMD1c_Genotyping_F1  (primers) | This paper | DKP-1 | GGGGAATATGCCACATTTAAGGAGA |
| sequence-based reagent | GRAMD1c_Genotyping_R1  (primers) | This paper | DKP-3 | GGTTTGTTTGAATTAGGAGTGGGTTG |
| sequence-based reagent | GRAMD1c_Stop_R  (primers) | This paper | LK219 | CGGGCCCGCGGTACCCTAGCTTTCAACAGCCATGCCAGTC |
| sequence-based reagent | GRAMD1c-sgRNA#1_AS  (primers) | This paper | TNP-28 | AAACAGGGGTAGATACTACATCTAC |
| sequence-based reagent | GRAMD1c-sgRNA#1_S  (primers) | This paper | TNP-27 | CACCGTAGATGTAGTATCTACCCCT |
| sequence-based reagent | GRAMD1c-sgRNA#2_AS  (primers) | This paper | TNP-30 | AAACACACTATAGTCCTTAATAGTC |
| sequence-based reagent | GRAMD1c-sgRNA#2_S  (primers) | This paper | TNP-29 | CACCGACTATTAAGGACTATAGTGT |
| sequence-based reagent | GRAMD3_F  (primers) | This paper | DD007 | GCAGTCGACATGACTGAACTACAGCAAGATGTGG |
| sequence-based reagent | GRAMD3_Stop_R  (primers) | This paper | DD008 | CAGTCATGGTACCTCAGTCACCATTCTCAAGCAACTTCTG |
| sequence-based reagent | 5'_NheI_miRFP_S  (primers) | This paper | TNP-89 | GTCAGATCCGCTAGCGCCACCATGGTAGCAGGTCATGCCTCTGG |
| sequence-based reagent | 3'_NotI_miRFP_AS  (primers) | This paper | TNP-90 | CCGGAACCGCGGCCGCGCTCTCAAGCGCGGTGATCCGC |
| sequence-based reagent | 5'_NotI_FKBP_linker_S  (primers) | This paper | TNP-91 | CTCGAAGCGCGGCCGCGGGAGCAGGAGGAG |
| sequence-based reagent | 3'_KpnI_FKBP_linker_AS  (primers) | This paper | TNP-92 | TTGGCCGTGGTACCACTACCACCAGCACTACCACC |
| sequence-based reagent | ssDNA_StopKI_HR_GRAMD1a_V2  (primers) | This paper | TNP-24 | GTGGGCAGTGTAGAAGTAGTCCTGGTAGGGGATGCCCTGCGGATCCCGGGCCCGCGGTACCGAATTCGAAGCTTGAGCTCGAGATCTACTAGTTAATCAGTCAGCACCTCGGAGTCCACCACACACCCGCCGGCCTGGG |
| sequence-based reagent | 5'_XHOI_ORP9_NS  (primers) | This paper | TNP-123 | ACTCAGATCTCGAGCTATGGTAGAATCAATTAAACACTGCATTG |
| sequence-based reagent | 3'_HINDIII_STOP_ORP9_CAS  (primers) | This paper | TNP-124 | TACCGAATTCGAAGCTTCTAATGCTTGGCAGCACCAAGACGTTTC |
| sequence-based reagent | 5'_HINDIII_OSBP_NS  (primers) | This paper | TNP-129 | CTCGAGCTCAAGCTTCGATGGCGGCGACGGAGCTGAGAGGAGTGG |
| sequence-based reagent | 3'_BAMHI_STOP_OSBP_CAS  (primers) | This paper | TNP-130 | AGATCCGGTGGATCCTCAGAAAATGTCCGGGCATGAGCTCCAGTC |
| sequence-based reagent | 5'_OSBP_SEQ_FW_2  (primers) | This paper | TNP-161 | AAGTCACTACAGTATGAAAGAGACC |
| sequence-based reagent | 5'_ORP4_SEQ_FW_1  (primers) | This paper | TNP-162 | CTGAAGTTAGATGACCTCAGCACG |
| sequence-based reagent | BSRG1-D4_E.COLI-BAMHI)  (gBlock) | This paper | TNG-3 | GACGAGCTGTACAAGTCCGGAAAGGGAAAAATAAACTTAGATCATAGTGGAGCCTATGTTGCACAGTTTGAAGTAGCCTGGGATGAAGTTTCATATGACAAAGAAGGAAATGAAGTTTTAACTCATAAAACATGGGATGGAAATTATCAAGATAAAACAGCTCACTATTCAACAGTAATACCTCTTGAAGCTAATGCAAGAAATATAAGAATAAAAGCAAGAGAGTGTACAGGCCTTGCTTGGGAATGGTGGAGAGATGTTATAAGTGAATATGATGTTCCATTAACAAATAATATAAATGTTTCAATATGGGGAACAACTTTATACCCTGGATCTAGTATTACTTACAATGCGGATCCGAATTCGAG |
| sequence-based reagent | ORP4_FRAG_1_XHOI  (gBlock) | This paper | TNG-8 | GAGGGTCCGGACTCAGATCTCGAGCTCAAGCTTCGATGGGTAAAGCAGCAGCTCCAAGTCGAGGAGGAGGATGTGGAGGTCGCTCCCGCGGGCTCTCGTCGCTGTTCACGGTTGTCCCCTGCCTGTCGTGTCATACAGCAGCACCAGGAATGAGTGCTTCAACATCAGGATCAGGACCAGAACCAAAACCACAACCACAACCAGTGCCCGAACCGGAGCGGGGACCGCTGTCAGAACAGGTGTCGGAGGCAGTTTCGGAGGCAGTGCCAAGATCGGAACCTGTGTCCGAGACGACGTCTGAGCCGGAGCCAGGGGCTGGGCAGCCATCGGAACTGCTGCAGGGGTCGCGGCCGGGGTCAGAGTCAAGCTCAGGTGTAGGGGCTGGGCCCTTCACTAAGGCCGCATCGGAGCCGCTCTCCCGGGCGGTGGGGAGCGCGACCTTTCTCAGACCCGAGTCAGGATCGCTGCCAGCGTTAAAGCCCCTGCCTCTTCTGCGACCAGGACAGGCGAAGACTCCTCTTGGGGTTCCAATGTCGGGGACTGGCACGACCTCCAGTGCCCCACTGGCCTTACTGCCTCTGGACAGCTTCGAGGGCTGGCTTCTCAAGTGGACCAACTATCTGAAGGGCTACCAGCGCCGCTGGTTCGTGCTGGGCAATGGTTTGCTCTCTTACTACAGAAATCAGGGTGAAATGGCCCACACGTGCCGTGGAACCATCAACCTGTCCACCGCGCACATTGACACGGAGGACTCTTGTGGTATCTTGCTGACCAGTGGGGCCAGGAGCTACCACCTCAAGGCCAGCTCAGAGGTGGACCGGCAGCAGTGGATCACCGCCCTGGAGCTGGCCAAGGCCAAGGCTGTCCGCGTGATGAACACTCATTCAGATGACTCTGGGGACGACGACGAGGCTACCACCCCAGCCGACAAGAGCGAGCTGCACCACACCCTGAAGAATCTTTCCCTGAAGTTAGATGACCTCAGCACGTGCAATGACCTCATCGCCAAGCACGGCGCCGCACTCCAGCGCTCCCTGACAGAGCTGGACGGCCTCAAGATCCCATCTGAGAGTGGGGAGAAGCTGAAGGTGGTGAATGAGCGGGCCACCCTCTTCCGCATCACATCCAATGCTATGATCAACGCCTGCAGGGACTTCTTGGAACTAGCAGAGATACACAGTCGGAAATGGCAGCGGGCACTGCAGTATGAGCAGGAGCAGCGCGTGCACTTGGAGGAAACCATTGAGCAGCTGGCGAAGCAGCACAACAGCCTCGAGCGGGCCTTCCACAGTGCCCCTGGCCGGCCGGCCAACCCCTCCAAGAGCTTCATTGAGGGAAGCCTCTTGACTCCCAAAGGAGAGGACAGTGAGGAAGATGAAGATACCGAGTACTTTGATGCCATGGAAGACTCC |
| sequence-based reagent | ORP4_FRAG_2_BAMHI  (gBlock) | This paper | TNG-9 | TGATGCCATGGAAGACTCCACATCCTTCATCACCGTGATCACCGAGGCCAAGGAAGACAGAAAAGCTGAAGGTAGCACCGGGACAAGTTCCGTGGACTGGAGCTCAGCAGACAATGTACTAGATGGTGCCTCGCTCGTGCCCAAGGGTTCATCCAAAGTCAAGAGGCGAGTCCGCATTCCCAACAAGCCCAACTACAGCCTTAACCTCTGGAGCATCATGAAGAACTGCATCGGCCGGGAGCTCTCCAGGATCCCCATGCCGGTGAACTTCAATGAGCCCCTGTCCATGCTCCAGCGGCTGACAGAGGACCTGGAGTACCACCACCTGCTGGACAAGGCAGTGCACTGCACCAGCTCAGTGGAGCAGATGTGCCTGGTGGCCGCCTTCTCTGTGTCCTCCTACTCCACCACAGTGCACCGCATCGCCAAGCCCTTCAACCCCATGCTGGGGGAGACCTTCGAGCTGGACCGCCTCGACGACATGGGCCTGCGCTCCCTCTGTGAGCAGGTGAGCCACCACCCCCCCTCAGCTGCGCACTACGTGTTCTCCAAGCATGGCTGGAGCCTCTGGCAGGAGATCACCATCTCCAGCAAGTTCCGGGGAAAATACATCTCCATCATGCCGCTAGGTGCCATCCACTTAGAATTCCAGGCCAGTGGGAATCACTACGTGTGGAGGAAGAGCACCTCAACTGTTCACAACATCATCGTGGGCAAGCTCTGGATCGACCAGTCAGGGGACATCGAGATTGTGAACCATAAGACCAATGACCGGTGCCAGCTGAAGTTCCTGCCCTACAGCTACTTCTCCAAAGAGGCAGCCCGGAAGGTGACAGGAGTGGTGAGTGACAGCCAGGGCAAGGCCCATTACGTGCTGTCCGGCTCGTGGGATGAACAAATGGAGTGCTCCAAGGTCATGCATAGCAGTCCCAGCAGCCCCAGCTCTGACGGGAAGCAGAAGACAGTGTACCAGACCCTGTCAGCCAAGCTGCTGTGGAAGAAGTACCCGCTGCCGGAGAACGCGGAGAACATGTACTACTTCTCAGAGCTGGCCCTGACCCTCAACGAGCACGAGGAGGGCGTAGCGCCAACCGACAGCCGCCTGCGGCCCGACCAGCGGCTGATGGAGAAGGGCCGTTGGGACGAGGCCAATACCGAGAAGCAGCGGCTGGAGGAGAAGCAGCGCCTGTCGCGGCGCCGGCGGCTGGAGGCCTGCGGGCCGGGCAGCAGCTGCAGCTCGGAGGAAGAGAAGGAGGCGGATGCCTATACACCATTATGGTTTGAAAAAAGATTAGATCCTTTAACAGGAGAAATGGCTTGTGTATATAAAGGAGGTTATTGGGAGGCCAAGGAGAAGCAAGACTGGCATATGTGCCCCAACATCTTCTGAGGTACCGCGGGCCCGGGATCCACCG |
| sequence-based reagent | XHOI_STARD4_KPNI  (gBlock) | This paper | TNG-14 | TCCGGACTCAGATCTCGAGAAATGGAAGGCCTGTCTGATGTTGCTTCTTTTGCAACTAAACTTAAAAACACTCTCATCCAGTACCATAGCATTGAAGAAGATAAGTGGCGAGTTGCTAAGAAAACGAAAGATGTAACTGTTTGGAGAAAACCCTCAGAAGAATTTAATGGATATCTCTACAAAGCCCAAGGTGTTATAGATGACCTTGTCTATAGTATAATAGACCATATACGCCCAGGGCCTTGTCGTTTGGATTGGGACAGCTTGATGACTTCTTTGGATATTCTGGAGAACTTTGAAGAGAATTGCTGTGTGATGCGTTACACTACTGCTGGTCAGCTTTGGAATATAATTTCCCCAAGAGAATTTGTTGATTTCTCCTATACTGTGGGCTATAAAGAAGGGCTTTTATCTTGTGGTAATAATCTCAACATGTTATGCTTTTTTTCAGTATGGTTTAAATATTTGTGTTTTCTTGGCTGGATGTCTCTTTCCTTGTGAGGTACCGCGGGCCCGGGATCCACC |
