# Supplementary material for: Movement of accessible plasma membrane cholesterol by the GRAMD1 lipid transfer protein complex
Source: eLife. 2019 Nov 14;8:e51401. doi: 10.7554/eLife.51401 (PMC6905856; doi:10.7554/eLife.51401)
Supplement: Supplementary file 3. — Lipid compositions of liposomes used for lipid transfer assays. Moles% of lipids used for the acceptor and donor liposomes in FRET-based lipid transfer experiments are described. [file elife-51401-supp3.docx]

| **Reagent type (species) or resource** | **Designation** | **Source or reference** | **Identifiers** | **Additional information** |
| --- | --- | --- | --- | --- |
| Other | in vitro DHE transfer assay  Donor liposomes | This paper |  | 90% of 1,2-dioleoyl-sn-glycero-3-phosphocholine (DOPC); 10% of ergosta-5,7,9(11),22-tetraen-3ß-ol (DHE) |
| Other | in vitro DHE transfer assay  Acceptor liposomes | This paper |  | 97.5% of 1,2-dioleoyl-sn-glycero-3-phosphocholine (DOPC); 2.5% of 1,2-dioleoyl-sn-glycero-3-phosphoethanolamine-N-(5-dimethylamino-1-naphthalenesulfonyl) (DNS-PE) |
| Other | liposomes used to generate calibration curve | This paper |  | 0% DHE: 97.5% of 1,2-dioleoyl-sn-glycero-3-phosphocholine (DOPC); 2.5% of 1,2-dioleoyl-sn-glycero-3-phosphoethanolamine-N-(5-dimethylamino-1-naphthalenesulfonyl) (DNS-PE) |
| Other | liposomes used to generate calibration curve | This paper |  | 5% DHE: 92.5% of 1,2-dioleoyl-sn-glycero-3-phosphocholine (DOPC); 2.5% of 1,2-dioleoyl-sn-glycero-3-phosphoethanolamine-N-(5-dimethylamino-1-naphthalenesulfonyl) (DNS-PE); 5% of ergosta-5,7,9(11),22-tetraen-3ß-ol (DHE) |
| Other | liposomes used to generate calibration curve | This paper |  | 10% DHE: 87.5% of 1,2-dioleoyl-sn-glycero-3-phosphocholine (DOPC); 2.5% of 1,2-dioleoyl-sn-glycero-3-phosphoethanolamine-N-(5-dimethylamino-1-naphthalenesulfonyl) (DNS-PE); 10% of ergosta-5,7,9(11),22-tetraen-3ß-ol (DHE) |
| Other | liposomes used to generate calibration curve | This paper |  | 15% DHE: 82.5% of 1,2-dioleoyl-sn-glycero-3-phosphocholine (DOPC); 2.5% of 1,2-dioleoyl-sn-glycero-3-phosphoethanolamine-N-(5-dimethylamino-1-naphthalenesulfonyl) (DNS-PE); 15% of ergosta-5,7,9(11),22-tetraen-3ß-ol (DHE) |
